# CLDN5 identified as a biomarker for metastasis and immune infiltration in gastric cancer via pan-cancer analysis

**DOI:** 10.18632/aging.204776

**Published:** 2023-06-07

**Authors:** Lu Han, De-Jun Cui, Bo Huang, Qian Yang, Tao Huang, Guo-Yuan Lin, Shao-Jie Chen

**Affiliations:** 1 Department of Gastroenterology, Guizhou Provincial People’s Hospital, Guiyang, Guizhou Province, China; 2 Department of Infectious Diseases, Affiliated Hospital of Guizhou Medical University, Guiyang, Guizhou Province, China; 3 Department of Hepatobiliary Surgery, The Affiliated Hospital of Guizhou Medical University, Guiyang, Guizhou, China

**Keywords:** CLDN5, immune infiltrating, EMT, pan-cancer analysis, bioinformatics

## Abstract

Background: CLDN5 protein is essential for the formation of tight junctions in epithelial cells, and has been associated with epithelial-mesenchymal transition. Research has indicated that CLDN5 is associated with tumor metastasis, the tumor microenvironment, and immunotherapy in multiple types of cancer. Also, no comprehensive evaluation of the expression of CLDN5 and immunotherapy signatures through a pan-cancer analysis or immunoassay has been performed.

Methods: We explored CLDN5's differential expression, survival analysis and clinicopathological staging through the TCGA database, and then corroborated the expression of CLDN5 by utilizing the GEO (Gene expression omnibus) database. To analyze CLDN5 KEGG, GO, and Hallmark mutations, as well as TIMER for immune infiltration, GSEA was utilized with ROC curve, mutation, and other factors such as survival, pathological stage, TME, MSI, TMB, immune cell infiltration, and DNA methylation. Immunohistochemistry was used to assess CLDN5 staining in gastric cancer tissues and paracancerous tissues. Visualization was done with R version 4.2.0 (http://www.rproject.org/).

Results: According to TCGA database, CLDN5 expression levels differed significantly between cancer and normal tissues, and the GEO database (GSE49051 and GSE 64951) and tissue microarrays confirmed this result. Infiltrating cluster of differentiation 8+ (CD8+) T cells, CD4+ cells, neutrophils, dendritic cells, and macrophages revealed a correlation with CLDN5 expression. DNA methylation, TMB, and MSI are related to CLDN5 expression. Based on the ROC curve analysis, CLDN5 demonstrates outstanding diagnostic effectiveness for gastric cancer and is comparable to CA-199.

Conclusions: The findings suggest that CLDN5 is implicated in the oncogenesis of diverse cancer types, underscoring its potential significance in cancer biology. Notably, CLDN5 could have implications in immune filtration and immune checkpoint inhibitor therapies, however, further research is needed to confirm this.

## INTRODUCTION

Human cancers substantially contribute to the global burden of disease, and they are widely acknowledged as a global problem with no global cure. Cancer treatment options and efficacies vary considerably with the clinical stage and associated risk factors [1]. The field of immunotherapy has become important in antitumor treatments over the past decade [2]. Immune cells infiltrating tumors are the most likely targets for drugs that improve survival [3]. For the first-line management of cancer, chemotherapy based on HER2 expression is recommended [4]. However, the specific immune infiltration-related signature molecules of cancer remain poorly defined, hence, identifying immune interaction biomarkers and new immunotherapy targets may be crucial for the treatment of cancer.

In humans, Claudins (CLDNs) encode the protein of the remarkable tight junction (TJ) component, which ranges in size from 20-27 kDa [5]. They may function in the regulation of ion homeostasis in the stomach by facilitating selective permeability [5]. Thus, CLDN proteins play an essential role in humans and are associated with epithelial-to-mesenchymal transition (EMT). Previous studies have shown that CLDN family genes play a crucial role in ovarian cancer, lung cancer, and gastric cancer [5–7]. CLDN5, a member of the CLDN family, is a transmembrane protein and a component of the TJ strand. Son et al. found that CLDN5 harbored frameshift mutations and mutational intratumoral heterogeneity (ITH) in gastric cancer. Meanwhile, high microsatellite instability (MSI-H) exists in gastric cancer [8]. From this study, CLDN5 was found to be a promising biomarker of gastric cancer. However, CLDN5 has not received considerable attention in pan-cancer research.

CLDN5 expression, enrichment analysis, pathway analysis, and correlation with cancer prognosis in the Cancer Genome Atlas (TCGA) and other public databases were investigated in this study. In this report, the importance of CLDN5 in pan-cancer as a potential biomarker and a valuable immune filter has been analyzed. Across different cancer types, the focus has been on the potential connection between the expression of CLDN5, immune infiltration, tumor mutational burden (TMB), microsatellite instability (MSI), and diverse immune-related effects.

## RESULTS

### CLDN5 mRNA expression in pan-cancers

**Figure 1:**
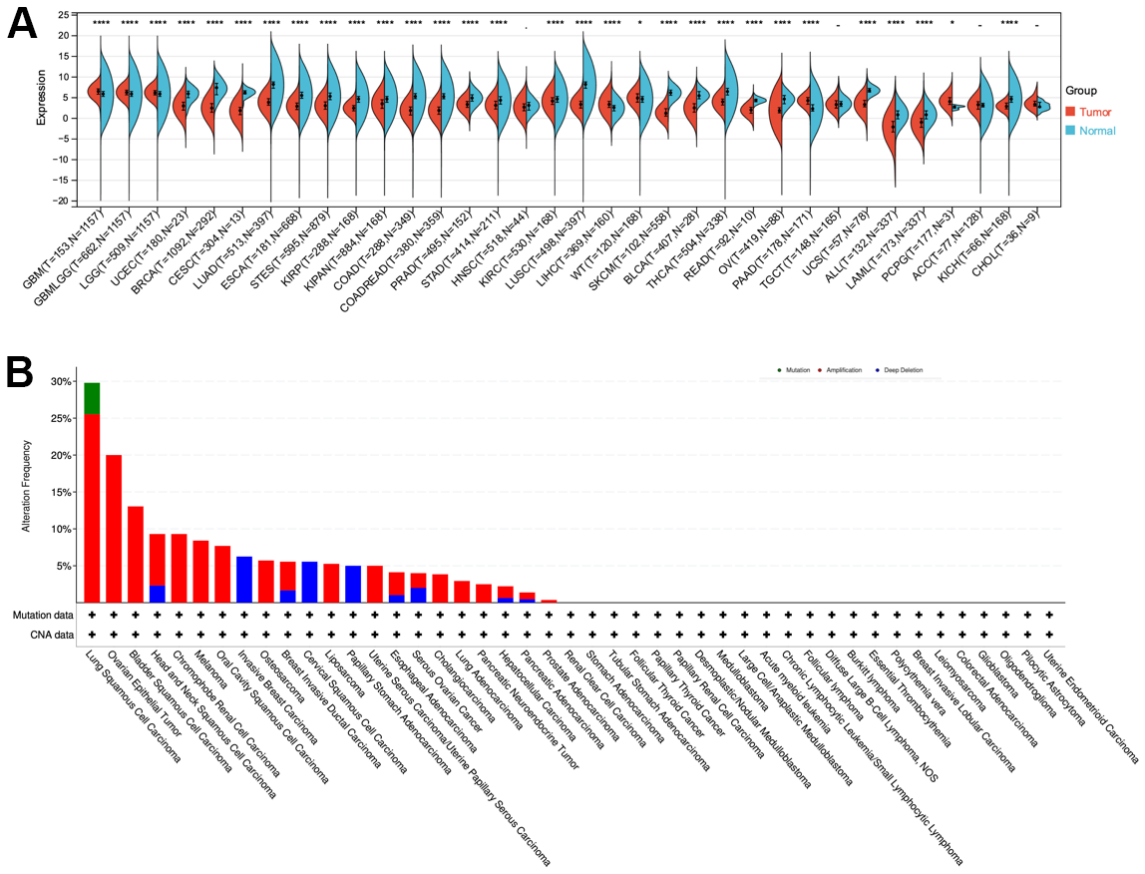
**CLDN5 alterations in pan-cancer.** (**A**) The results from TCGA database indicated that CLDN5 expression is reduced in most tumors. (**B**) Co-occurrence of genetic mutations with CLDN5 alterations in tumors.

The GTEx and TCGA databases were used to analyze CLDN5 expression in multiple tumor types and normal tissues. Significant upregulation of CLDN5 was observed in the following five tumors: glioblastoma multiforme (GBM), brain lower-grade glioma (LGG), liver hepatocellular carcinoma (LIHC), pancreatic adenocarcinoma (PAAD), and pheochromocytoma and paraganglioma (PCPG) (Figure 1A). Compared with the expression level in adjacent normal tissues, CLDN5 expression level was lower in 20 tumors, such as breast invasive carcinoma (BRCA), bladder urothelial carcinoma (BLCA), cervical squamous cell carcinoma and endocervical adenocarcinoma (CESC), colon adenocarcinoma (COAD), esophageal carcinoma (ESCA), head and neck squamous cell carcinoma (HNSC), kidney chromophobe (KICH), kidney renal papillary cell carcinoma (KIRP), kidney chromophobe (KIRC), lung adenocarcinoma (LUAD), lung squamous cell carcinoma (LUSC), acute myeloid leukemia (LAML), prostate adenocarcinoma (PRAD), rectum adenocarcinoma (READ), stomach adenocarcinoma (STAD), skin cutaneous melanoma (SKCM), thyroid carcinoma (THCA), ovarian serous cystadenocarcinoma (OV), uterine corpus endometrial carcinoma (UCEC), and uterine carcinosarcoma (UCS) (Figure 1A).

Additionally, CLDN5 mutations included missense, deletion, and amplification mutations among other nonsynonymous mutations. The CLDN5 gene was altered in 80 (3%) of 2565 patients with various tumors, according to cBioPortal. Patients with NSCLC had the highest frequency of CLDN5 mutations (28.26%), with amplification accounting for most of the mutations (11.82%, 545 of 4609; Figure 1B).

### CLDN5 expression and clinicopathology in pan-cancers

**Figure 2:**
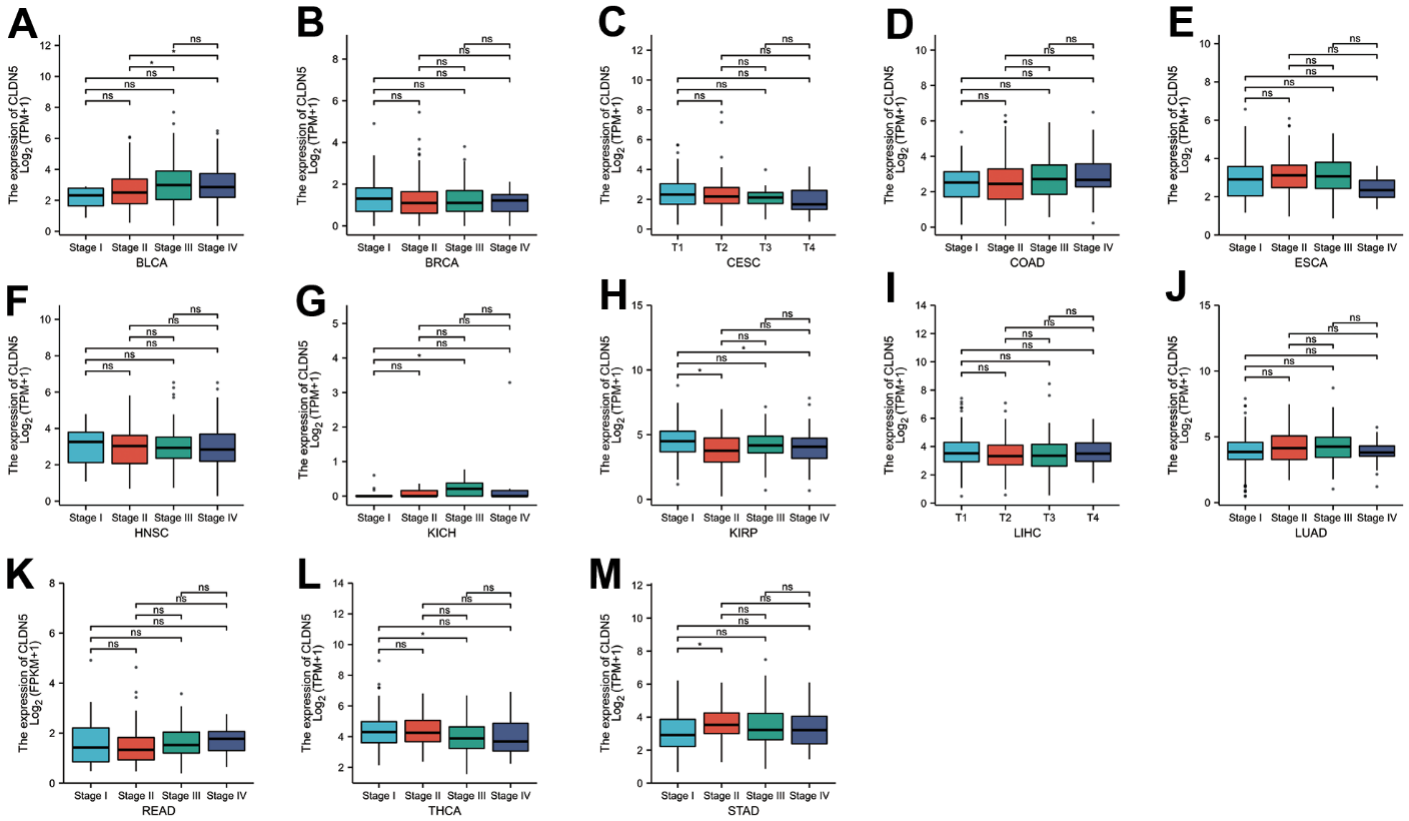
**The TCGA data were used to investigate correlations between CLDN5 expression and the major pathological stages including stage I, stage II, stage III, and stage IV.** (**A**–**E**) Correlations between CLDN5 expression and the major pathological stages of BLCA BLCA, BRCA, COAD and ESCA. (**F**–**J**) Correlations between CLDN5 expression and the major pathological stages of HNSC, KICH, KIRP, LIHC, LUAD. (**K**–**M**) Correlations between CLDN5 expression and the major pathological stages of READ, THCA, and STAD. For log scale, Log2 (TPM+1) was used. * p < 0:05, ** p < 0:01, and *** p < 0:001.

CLDN5 expression was studied in patients with stages I, II, III, and IV cancers, including the correlation between CLDN5 expression and clinicopathological features in various cancers. CLDN5 expression was found in a variety of advanced cancers, including BLCA, BRCA, CESC, COAD, ESCA, HNSC, LIHC, LUAD, KIRP, and READ (Figure 2A–2M). Particularly, CLDN5 expression was significantly higher in stage II than in stage I STAD, suggesting that elevated CLDN5 may be associated with the progression of disease in patients diagnosed with STAD (Figure 2M).

### Pan-cancer analysis of the multifaceted prognostic value of CLDN5

**Figure 3:**
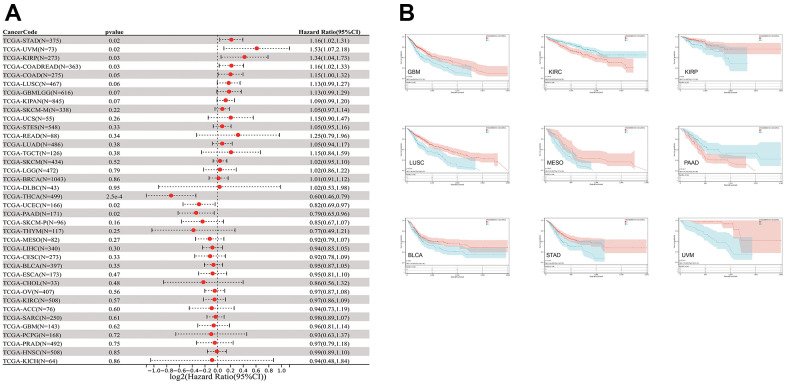
**The relationship between CLDN5 expression and cancer patients' OS.** (**A**) A forest plot of CLDN5 hazard ratios in 33 multiple cancers. (**B**) Kaplan-Meier survival curves of OS for patients groups defined by CLDN5 expression in GBM, KIRP, COADREAD, STAD, KIRC, LUSC, BLCA, MESO, UVM, PAAD, PCPG, and KICH.

The relationship between CLDN5 expression and patient prognosis using a pan-cancer dataset was analyzed. The survival metrics included OS, DSS, DFI, and PFI. Based on the Cox regression analysis of 33 cancers (Figure 3A and Supplementary Figures 1–3), in seven cancers, including STAD, uveal melanoma (UVM), KIRP, COAD, THCA, UCEC, and PAAD, CLDN5 expression was significantly correlated with OS. Upregulated CLDN5 expression was found to be strongly correlated with poor OS in PAAD, PCPG, and KICH. Meanwhile, downregulated CLDN5 in BLCA, COAD, GBM, KIRC, KIRP, STAD, LUSC, UVM, and MESO was linked with poor OS (Figure 3B).

**Figure 4:**
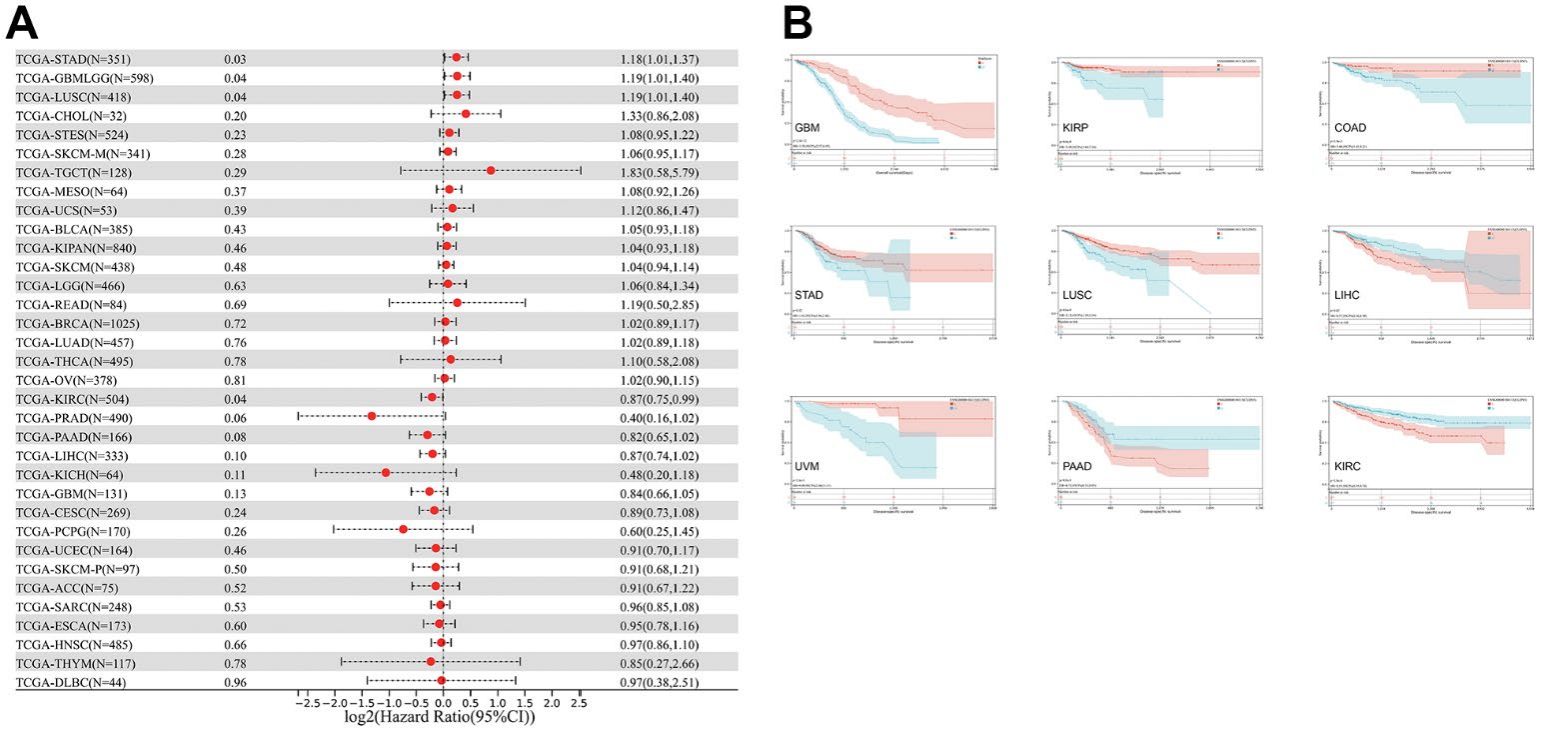
**The relationship between CLDN5 expression and cancer patients' DSS.** (**A**) A forest plot of CLDN5 hazard ratios in 33 multiple cancers. (**B**) Kaplan-Meier survival curves of DSS for patients groups defined by CLDN5 expression in GBM, KIRP, COAD, STAD, LUSC, LIHC, UVM, PAAD, and KIRC.

The relationship between CLDN5 expression and DSS was also evaluated. CLDN5 expression influenced DSS in the following seven cancer types (Figure 4A): COAD, KIRP, LUSC, UVM, STAD, GBMLGG, and KIRC. Increased CDN5 expression was associated with poor DSS in patients with LIHC and PAAD, whereas decreased CDN5 expression was associated with poor DSS in patients with COAD, GBM, KIRC, KIRP, LUSC, STAD, and UVM (Figure 4B).

A study of CLDN5 expression in patients with KIRP, HNSC, and THCA found that CLDN5 expression affected DFI (Supplementary Figure 1A). Based on the Kaplan-Meier DFI curves, increased expression of CDN5 mRNA was associated with an unfavorable DFI in THCA, while an unfavorable DFI was reduced in HNSC and KIRP (Supplementary Figure 1B).

Cox regression analysis of PFI indicated that CLDN5 expression influenced eight types of cancers, such as STAD, UVM, KIRP, COAD, THCA, UCEC, and PAAD (Supplementary Figure 2A). According to the findings of the Kaplan-Meier analysis, increased CLDN5 expression was associated with a poor prognosis in four types of cancers, namely, COAD, GBM, KIRP, STAD, LUSC, and UVM (Supplementary Figure 2B).

### CLDN5 expression and immune infiltration in pan-cancer research

**Figure 5:**
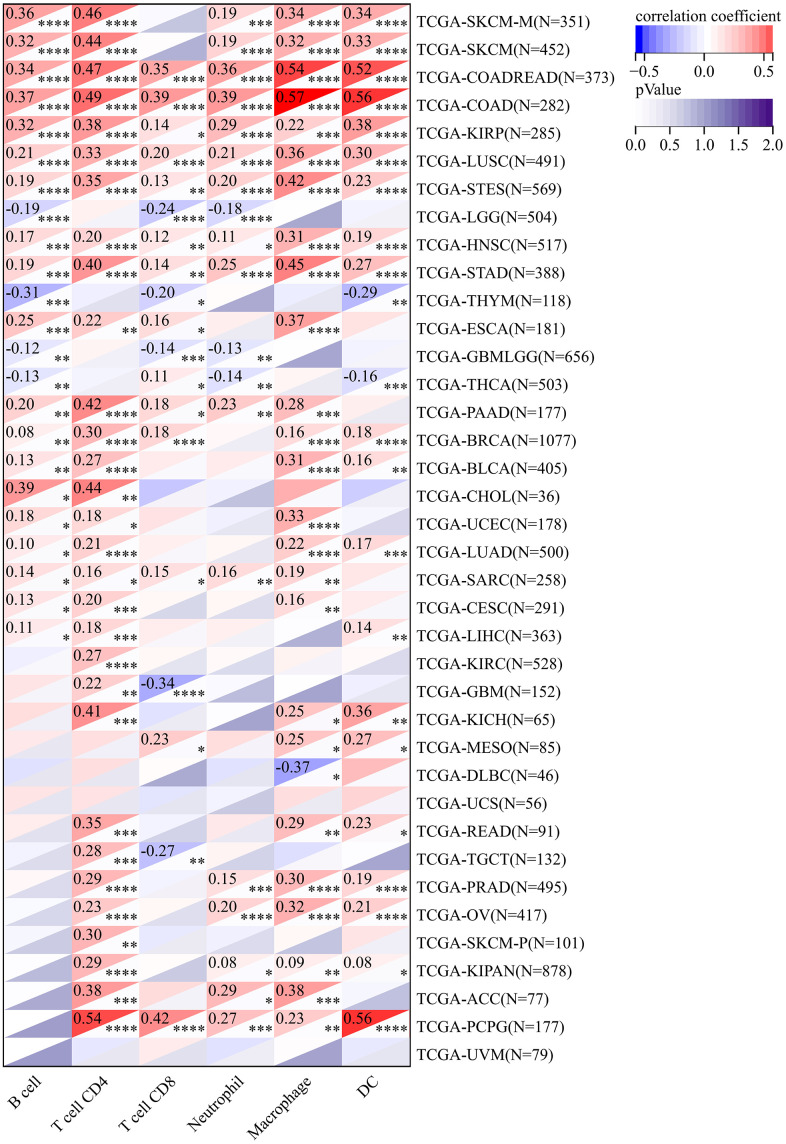
**In the TIMER database, CLDN5 expression was found to be significantly correlated with the levels of infiltration of various immune cells.** * p < 0:05, ** p < 0:01, and *** p < 0:001.

The TIMER database was used to analyze the association between CLDN5 expression and immune infiltration levels due to its unique relationship with immune response. CLDN5 expression was linked with an abundance of infiltrating immune cells in eight cancer types, CD4+ T cells in 30 different cancer types, CD8+ T cells in 19, macrophages in 26, neutrophils in 19, and DCs in 22 types of cancer (Figure 5), including COAD, HNSC, kidney renal clear cell carcinoma (KIRC), and LIHC (Figure 5). In particular, CD8+ T cell infiltration was also positively correlated with high CLDN5 expression in BLCA, BRCA, KIRP, brain LGG, LIHC, PAAD, PCPG, PRAD, READ, testicular germ cell tumor (TGCT), thymoma (THYM), UCEC, and UVM (P<0.05).

**Figure 6:**
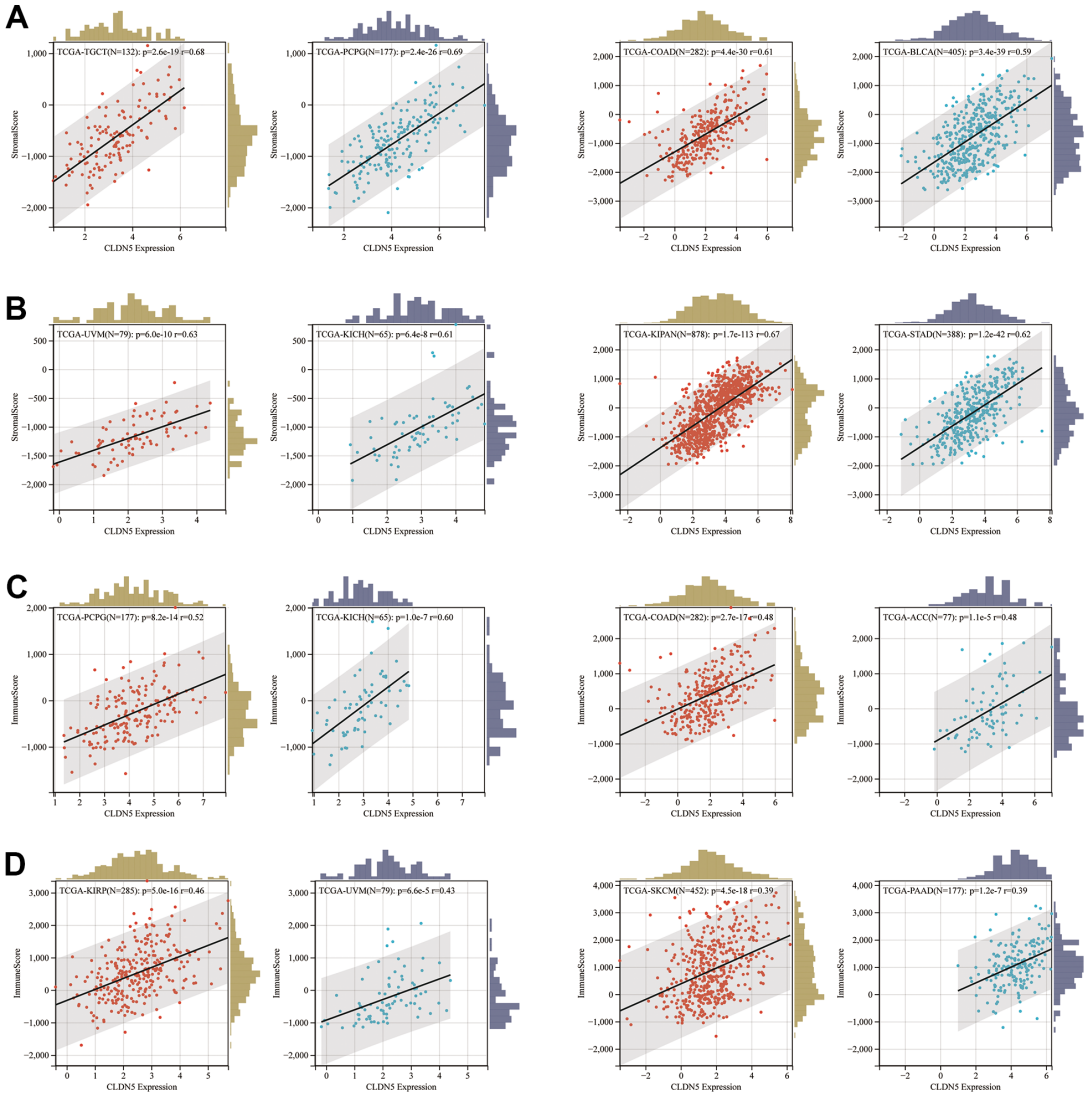
The positive association between CLDN5 expression and stromal score (**A**, **B**) and immune score (**C**, **D**) in top 8 tumors.

The immune and stromal cells scored higher in CLDN5 expression in KIRC, COAD, UVM, KIRP, and READ tissues (Figure 6), indicating that CLDN5 had a strong positive correlation with the ESTIMATEScore (Supplementary Figure 3).

**Figure 7:**
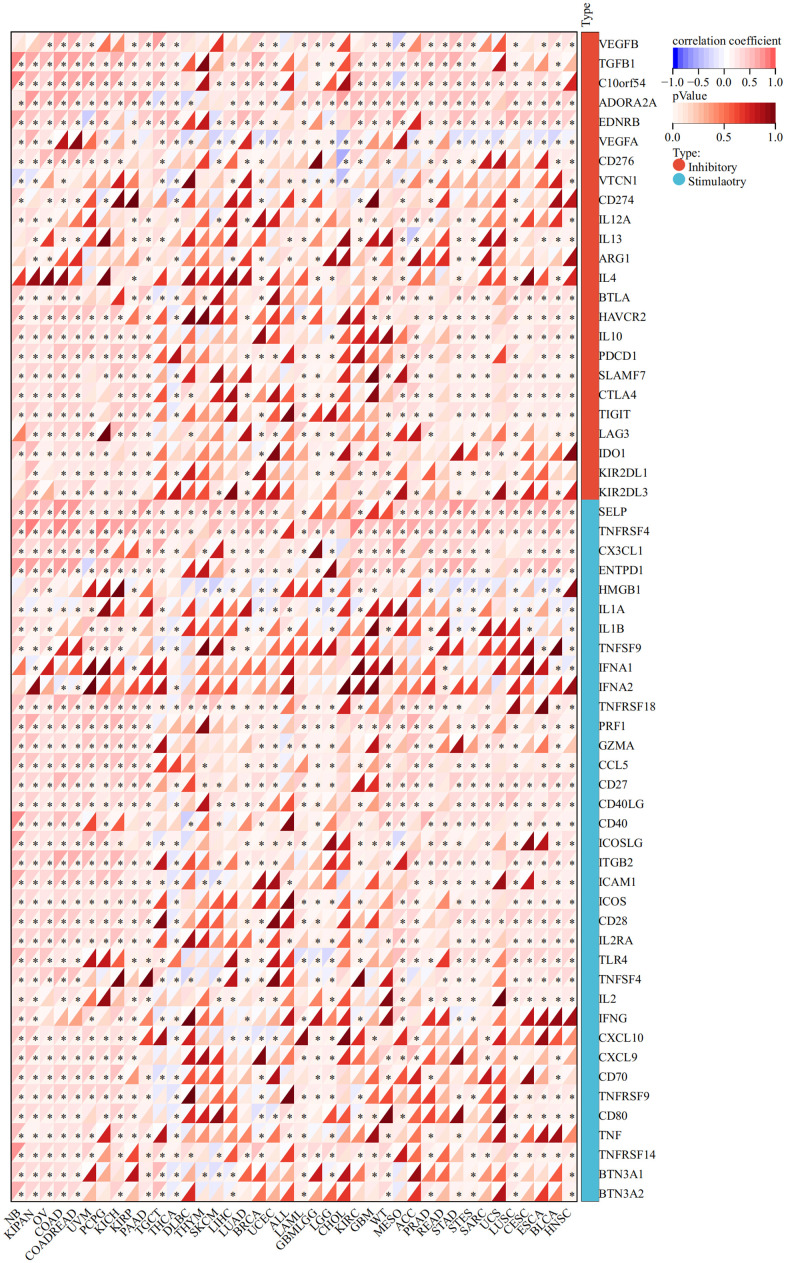
The association heatmaps between CLDN5 expression and immune checkpoints genes expression in 33 tumors *P < 0.05, **P < 0.01, ***P < 0.001.

The expression of CLDN5 was strongly linked with that of immune checkpoint genes, such as TGFβ1, C10orf54, and ADORA2A in most tumor types, including STAD, COAD, and ESCA (Figure 7).

### Mutational analysis of CLDN5 in various tumors

**Figure 8:**
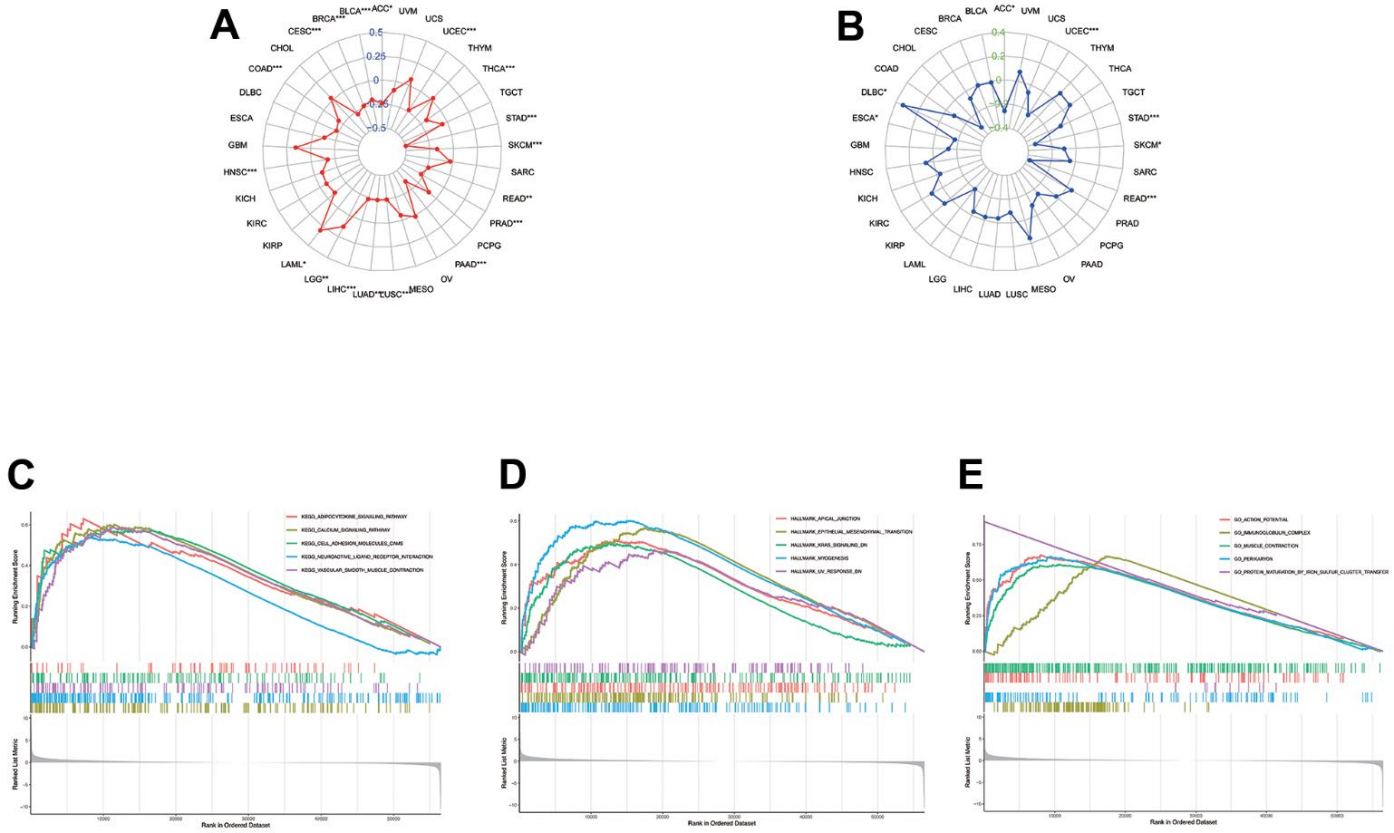
The association between CLDN5 among TMB levels (**A**) and MSI (**B**). (**C**–**E**) KEGG, Hallmark, and Go analysis of CLDN5 in STAD. (**C**) KEGG analysis of CLDN5 in STAD. (**D**) Hallmark analysis of CLDN5 in STAD. (**E**) GO analysis of CLDN5 in STAD.

MSI and TMB are the two new immunotherapy response biomarkers. The relationship between CLDN5 expression and TMB was investigated. CLDN5 expression was found to be associated with TMB in a variety of tumors, including ACC, UCEC, THCA, STAD, SKCM, READ, PRAD, PAAD, LUSC, LUAD, LIHC, LGG, LAML, HNSC, COAD, CESC, BRCA, BLCA. CLDN5 expression was found to be positive in the presence of LAML and negative in the presence of COAD, STAD, THCA, PRAD, PAAD, BRCA, BLCA, CESC, and LIHC (Figure 8A). CLDN5 expression was also linked to MSI in 33 different cancers, including ACC, DLBC, ESCA, READ, STAD, and UCEC, with positive correlations for DLBC and negative correlations for STAD, READ, UCEC, and ACC (Figure 8B). Meanwhile, the mutation sites and types of CLDN5 in 15 tumors were also analyzed. The results implied that the major mutations in CLDN5 are frameshift, missense, and deletions in many sites of most cancers, indicating that mutations of CLDN5 are likely to be associated with cancers. This suggests that CLDN5 might be relevant to subsequent treatment (Supplementary Figure 4).

### Association of CLDN5 with DNA methylesterase

**Figure 9:**
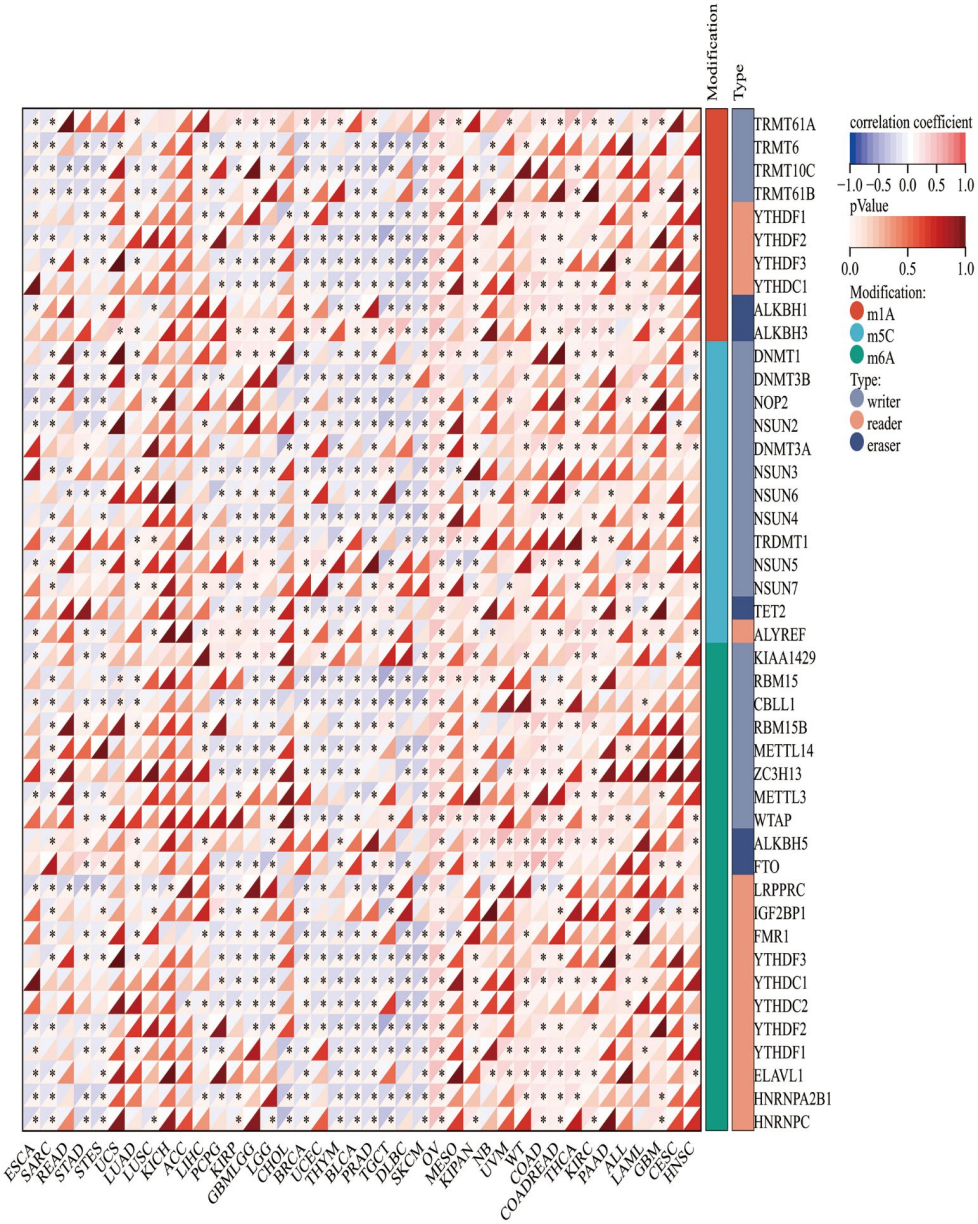
The association heatmaps between CLDN5 expression and DNA methylesterase genes expression in 33 tumors *P < 0.05, **P < 0.01, ***P < 0.001.

Since CLDN5 RNA expression was reduced in tumors compared with that in adjacent tissues, an investigation into the correlation with both CLDN5 and DNA methylation enzymes was conducted. CLDN5 RNA expression was negatively linked with a majority of methylation enzymes, particularly BRCA, BLCA, and PRAD (Figure 9).

### Functional enrichment analysis of CLDN5 in pan-cancer

Based on the available results, a close relationship between CLDN5 and STAD was found, as GSEA was utilized to understand the regulation of CLDN5 in STAD. The top five significant KEGG terms were adipocytokine signaling pathway, calcium signaling pathway, cell adhesion, neuroactive ligand-receptor interaction, and vascular smooth muscle contraction, all of which were associated with CLDN5 (Figure 8C). GO analysis was also performed on the CLDN5 genes and several enriched GO categories, including action potential, immunoglobulin complex, muscle contraction, perikaryon, and protein maturation via iron-sulfur cluster transfer, were discovered (Figure 8D). Hallmark terms were associated with lower CLDN5 expression in apical junction, epithelial-mesenchymal transition, KRAS signaling, myogenesis, and UV (Figure 8E).

### Validation of CLDN5 expression in the GEO datasets, tumor tissue microarrays, and ROC curve analysis

**Figure 10:**
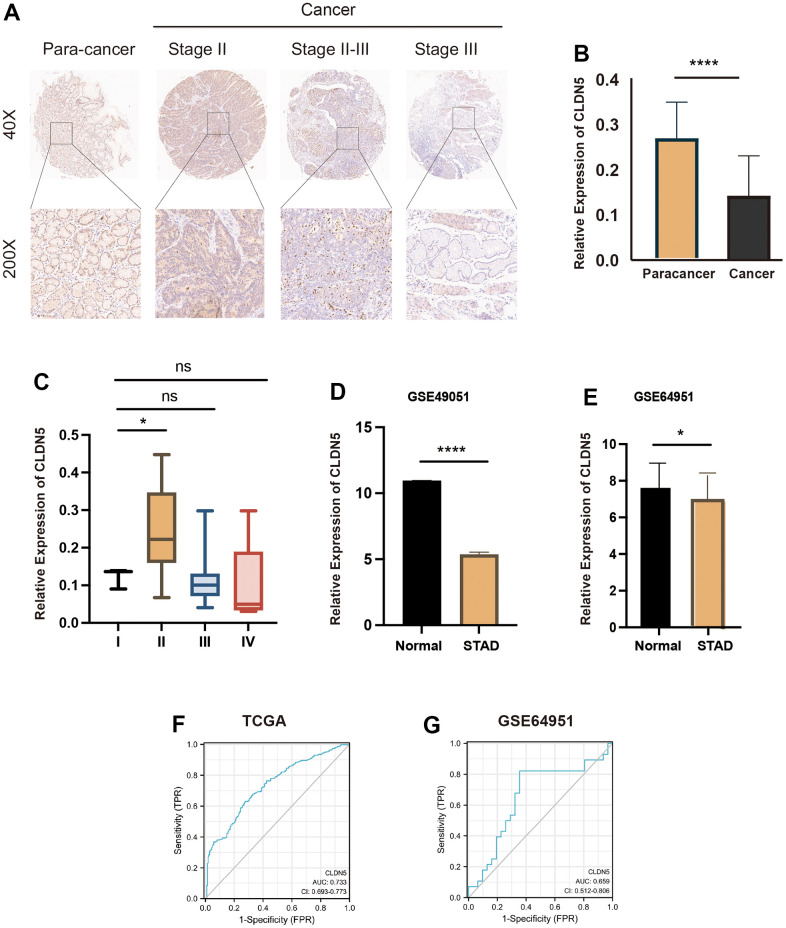
**CLDN5 staining in gastric adenocarcinoma tissue microarrays using immunohistochemical methods.** (**A**) CLDN5 is highly expressed in the para cancer and decreases with change in the pathological stage. (**B**) Relative expression of CLDN5 in 78 pairs of STAD and para-cancerous tissues (p < 0.001). (**C**) Relationship between CLDN5 expression and clinical stage, CLDN5 was elevated in clinical stage II, consistent with TCGA results. (**D**) CLDN5 expression in GSE49051 datasets, CLDN5 expression was decreased in normal than STAD (p <0.05). (**E**–**G**) Validation of CLDN5 expression in GEO datasets and ROC curves analysis. (**E**) CLDN5 expression in GSE64951 datasets, CLDN5 expression was decreased in normal salivary than STAD salivary (p <0.05). (**F**) The TCGA training result. The AUC values were 0.733. (**G**) The GEO test sets results. The AUC values were 0.659.

A GEO dataset, GSE49051, was downloaded from the database to determine whether CLDN5 expression was related to tumor. There were significant differences in CLDN5 expression in tumorous tissues and normal tissues, as indicated by the results (P< 0.05) (Figure 10D). The salivary transcriptomic datasets (GSE64951) were downloaded for analysis to see if CLDN5 can be used as a biomarker for STAD. A statistically significant difference (P<0.05) in CLDN5 levels between STAD saliva and normal saliva was observed (Figure 10E). Further, CLDN5 immunohistochemical staining was performed using gastric adenocarcinoma tissue microarrays. The clinicopathological characteristics of 78 patients with STAD are listed in Table 1. The results suggested that CLDN5 expression was reduced in the tumor compared with that in para cancer, with a decrease in the mean optical density (P<0.01, Figure 10A, 10B). Statistics according to the pathological tissue stage and TNM stage were also performed. CLDN5 levels were found to be higher in clinical stage II compared with that in stage I (Figure 10C). The findings were statistically significant and corroborated the findings of TCGA, indicating elevated CLDN5 was associated with lymph node metastasis in patients with gastric cancer, with clinical staging being lymph node metastasis.

In addition, AUC values of 0.733 and 0.659 were found in the TCGA training and GEO test sets, respectively (Figure 10F, 10G). For predicting gastric cancer and immunotherapy, CLDN5 was found to be a promising biomarker of STAD.

**Table 1:** Basic clinical information sheet.

**Clinical characteristic**	**Variable**	**Number of patients (n=80)**
Age	>=60	51
	<60	29
Gender	Female	17
	Male	63
Pathologic stage	II	26
	III	25
	II-III	29
Stage T	1	1
	2	7
	3	45
	4a+4b	27
Stage N	0	17
	1	14
	2	22
	3a+3b	26
Stage M	0	73
	1	7
Clinical stage	I	5
	II	22
	III	46
	IV	7

## DISCUSSION

Cancer mortality rates are rapidly increasing worldwide, and it is now the major cause of death across all countries [9]. Female breast carcinoma is the most prevalent type of cancer, accounting for approximately 2.3 million new cases each year (11.7%). Lung (11.4%), colorectal (10.0%), prostate (7.3%), and stomach (5.6%) cancers are the most prevalent [10]. Lung, colorectal, liver, stomach, and female breast cancer have the highest mortality rates [10]. Surgical resection, radiotherapy, and adjuvant chemotherapy are the most common cancer treatments, but their effectiveness is limited [11]. Cancer cell metastasis seems to have a significant impact on the disease prognosis, with a significantly lower 5-year survival rate than stabilized tumors [12]. The pan-cancer analysis provides a significant overview of the evolution of cancer prevention and treatment strategies. We determined that CLDN5 was associated with the prognosis of most tumours by difference-in-difference and survival analyses. Further we found that CLDN5 was associated with gastric cancer and immune infiltration by MSI and TMB, methylation enzymes as well as immune infiltration analysis and immune checkpoint analysis. We then performed GSEA analysis and the results suggested that CLDN5 was associated with immunotherapy. Finally we verified that CLDN5 is reduced in gastric cancer by multiple datasets and tissue microarrays, and our results suggest that CLDN5 is a promising molecule for the early diagnosis and treatment of gastric cancer.

Numerous studies have shown that TJ proteins are closely related to tumor cell invasion and metastasis [13]. CLDN5 is an endothelial cell-specific component of TJ strands that was first reported by Morita et al. [5]. The reduction of CLDN5 has increased the progression of glioma cells and the leakage of the brain-blood barrier (BBB) [14, 15]. SIRT1 and KLF4 inhibit the migration and invasion of ovarian cancer cells by activating the transcription of CLDN5. According to previous studies, it appears to be closely related to tumor metastasis [6]. Results suggested that low doses of bevacizumab can increase CLDN5 expression to alleviate lung cancer metastasis, suggesting that CLDN5 is associated with anti-tumor immunotherapy [7].

In this study, we present the first comprehensive analysis of CLDN5 and pan-cancer, including prognosis, immune infiltration, methylation and mutation. The expression of CLDN5 in most tumors was simultaneously associated with prognosis, including BLCA, BRCA, CESC, COAD, ESCA, HNSC, KICH, and KIRP. In several cancers, lower CLDN5 expression was associated with a worse OS, DSS, DFI, or PFI. (Figures 2, 3 and Supplementary Figures 1, 2), especially in STAD, COAD, KIRP, and LUSC. CLDN5 expression and immune infiltration were found to be strongly linked to various types of cancers. (Figures 5, 7). The results suggest that CLDN5 can be used as a biomarker for tumor diagnosis and prognostic impact.

Due to the reduced expression of CLDN5 in tumors when compared with that in normal tissues, further examination of the mutation of CLDN5 from previous reports in the literature and this analysis in TCGA database was conducted. In this study, CLDN5 mutations were discovered as a variety of nonsynonymous mutations, including amplifications and deep deletions. Recent research suggested that CLDN5 frameshift mutations in TJ and gap junction genes may cause gastric cancer. Multiple mutations typically precede CLDN5 amplification or deep deletions according to the molecular time analysis, which might be supported by a previous study. This suggests that mutations in CLDN5 are key to tumorigenesis [7]. To further identify potential mutations of CLDN5 in pan-cancer, a TMB analysis was conducted. Surprisingly, a strong negative correlation between TMB and CLDN5 expression in STAD, COAD, READ, PAAD, and BLCA was found; meanwhile, a positive correlation was associated with LAML. As TMB level is commonly regarded as a remarkable biomarker associated with treatment effects in many immune checkpoint inhibitor (ICI)–treated tumors, CLDN5 mutation predicts the therapeutic efficacy of ICIs across tumor types [16]. These findings suggest that CLDN5 is significantly involved in tumor immune evasion.

To investigate the role of CLDN5 in immune infiltration further, CLDN5 expression was analyzed, which revealed a positive correlation between CD4+ T cells and macrophage cells in most tumors. However, further research must be conducted to understand the relationship between CLDN5 and CD4+ T cells and macrophage cells. According to the current findings, CLDN5 has a strong association with stromal and immune scores, and CLDN5 expression is strongly correlated with the estimated score in most cancer types.

DNA mismatch repair deficiency (MMRD) causes MSI [17, 18]. Microsatellites (MS) are short tandem repeats (1-6 nucleotides long) found throughout the genome and are highly susceptible to mutation [19]. MSI cells accumulate a high number of frameshift mutations [20]. Frameshift mutations in cancer-related genes may promote tumorigenesis and, as a result, are shared by MSI tumors that arise independently [18]. The results showed CLDN5 expression was negatively associated with UCEC, STAD, and READ and positively correlated with DLBC, suggesting that MSI-H status is found in patients with low CLDN5 expression. Combined with the previous differential expression, survival analysis, MSI and TMB results, and immune cell infiltration analysis, CLDN5 expression was found to be strongly correlated with prognosis and treatment in patients with STAD.

Methylation of CpG island in the promoters of tumor-related genes, which is significantly correlated with clinical behavior in many cancers, is a canonical observation in many cancers [21]. The occurrence and progression of various tumors are linked to gene methylation [22]. The result suggests that CLDN5 was negatively associated with a majority of methylesterases in several tumor species, indicating that methylesterases (specifically m6A methylesterases) regulate CLDN5 transcription. However, the relationship between CLDN5 and methylation enzymes needs additional investigation.

Crucial signaling pathways of CLDN5 were then evaluated. CLDN5 is related to adipocytokine and cell adhesion molecules (CAMs). Hallmark analysis suggested that low expression of CLDN5 is related to EMT and KRAS signaling pathways. KRAS mutations are markers and predictors of inadequate efficacy and poor prognosis of bevacizumab in COAD [23]. CLDN5 appears to be linked to tumor metastasis and tumor immunotherapy, but further studies are required to confirm this. According to the KEGG analysis conducted in this study, CLDN5 was associated with an increase in many signaling pathways in correspondence with previous research (Figure 10A).

Meanwhile, CLDN5 expression in several datasets was validated, and ROC curves in TCGA training and the GEO test set were assessed, suggesting an effective role for CLDN5 in the diagnosis of gastric cancer. Interestingly, CA19-9 and carcinoembryonic antigen, two markers regarded as valuable for early-stage GC diagnosis, had AUCs of 0.73 and 0.68, respectively [24]. The findings indicated that the diagnostic efficacies of CLDN5 and CA-199 are equivalent. Meanwhile, the differences in CLDN5 expression in gastric adenocarcinoma and paraneoplastic tissues are also consistent with our previous analysis.

This study contributes to the development of CLDN5-targeting therapeutic strategies by bringing attention to the use of CLDN5 as a possible prognostic biomarker in a variety of cancers in the context of immuno-oncology.

In summary, the results of the current study demonstrate that CLDN5 gene variants and expression are linked to clinical outcomes, and CLDN5 genetic changes may serve as biomarkers in forecasting the effects of immunotherapy. As a result, they may become promising biomarkers for predicting ICI response in a variety of tumors. However, the limitations of the study necessitate the need for additional research.

## CONCLUSIONS

In summary, the prevailing research suggests that CLDN5 is related to immune infiltration, MSI, TMB, and DNA methylation in various cancers, as well as the outcomes of cancer patients and immune infiltration across diverse cancers, particularly STAD. CLDN5 was found to be strongly associated with immune gene expression in a variety of cancers. CLDN5 may also find use as a prognostic biomarker.

## MATERIALS AND METHODS

### The expression of CLDN5 in pan-cancer

The expression levels of CLDN5 in cancer and normal tissues were compared using data from TCGA and the GTEx databases [25]. The data included 33 different cancers, as well as RNA sequencing and clinical follow-up data. Log2 conversion was used to normalize microarray data.

### CLDN5 prognostic evaluation in pan-cancer

In 33 different types of cancer, forest plots and Kaplan-Meier curves were used to explore the relationship between CLDN5 expression and patient prognosis, including overall survival (OS), disease-specific survival (DSS), disease-free interval (DFI), and progression-free interval (PFI) [25]. Multivariate survival analysis was used to measure the hazard ratios (HRs) and 95% confidence intervals [25].

### CLDN5 immune infiltration assessment

To examine CLDN5 expression in six types of TIICs, neutrophils, B cells, macrophages, cluster of differentiation 4+ (CD4+) T cells, CD8+ T cells, and dendritic cells (DCs) were analyzed. Results were presented as ImmuneScores, StromalScores, and ESTIMATEScores. Higher ImmuneScores or StromalScores indicated a higher immune or stromal ratio, and an increase in the respective score would result in a larger ratio. The ESTIMATEScore was calculated by combining the proportions of both components in the tumor microenvironment (TME) of multiple cancers.

A correlation between immune checkpoint–related genes and CLDN5 gene expression was observed in multiple cancers according to Spearman correlation analysis. Heat maps display cancer types along the horizontal axis, immune scores along the vertical axis, and correlation scores (P<0.05, P<0.01, P<0.001).

### CLDN5 mutation and methylation enzyme investigation in pan-cancer

In this study, the link between m1A, m5C, and m6A-related gene expression and CLDN5 expression was investigated. Pearson’s correlation was used in monitoring the correlation of methyltransferases with CLDN5 expression. Gene expression data were obtained from TCGA and cBioPortal. The Simple Nucleotide Variation dataset for level 4 of all TCGA samples was obtained from GDC (https://portal.gdc.cancer.gov/) and processed using the MuTect2 software [26]. Mutation data from the samples were integrated using the R package, maptools (version 2.2.10).

### Relationship between CLDN5 gene expression and TMB or MSI

Spearman's correlation analysis was used to examine the relationship between CLDN5 expression and TMB or MSI data from TCGA [25]. CLDN5 expression data corresponded to TMB or MSI on the horizontal axis, cancer types on the ordinate, and cancer sizes on the horizontal axis.

### Gene set enrichment analysis (GSEA) analyses of CLDN5

The biological and molecular functions of CLDN5 in stomach adenocarcinoma (STAD) were examined using the KEGG analysis. GSEA was used to search into the possible molecular mechanisms of CLDN5 in STAD. ClusterProfiler, an R package, was used to perform KEGG and GSEA.

### Validation of CLDN5 expression in the GEO database and tissue microarrays

The GSE49051 dataset was acquired for validating CLDN5 expression in STAD. In addition, messenger RNA (mRNA) sequencing data obtained from the saliva of patients with STAD and normal patients for differential expression validation (GSE64951) were downloaded. Further, CLDN5 was validated for its diagnostic effectiveness. Primary human gastric cancer tissue microarray data (HStmA160CS01) were obtained from Outdo Biotech (Shanghai, China), comprising 80 gastric cancer and 80 corresponding adjacent normal samples. To analyze the differential expression of CLDN5, Image Pro Plus and GraphPad Prism v9.0 were used.

### Immunohistochemistry

Dewaxed and rehydrated paraffin-embedded sections were incubated with primary antibodies specific to CLDN5 (1:100, ZhengNeng) at 4° C overnight and biotin-labeled secondary antibodies at 37° C for 1 hour. The slides were then made dirty with DAB and stained with hematoxylin. Following this, the slides were examined under a microscope (Leica, Wetzlar, Germany).

### Statistical analysis

Statistical analysis was performed using the R language software (version 4.2.0) (https://www.r-project.org) and GraphPad Prism v9.0. ANOVA and Wilcoxon's test were used to analyze differences between two and more groups, respectively. A log-rank test and Kaplan-Meier explanation were used to estimate the difference in OS between groups. The subtypes, clinicopathological features, risk scores, immune checkpoint expression, methyltransferases, and levels of immune infiltration were determined using Pearson's correlation test. The results were found to be statistically significant (P<0.05).

### Data availability statement

The datasets shown in this study are available in online repositories.

## Supplementary Material

Supplementary Figures
