# Supplementary material for: CLDN5 identified as a biomarker for metastasis and immune infiltration in gastric cancer via pan-cancer analysis
Source: Aging (Albany NY). 2023 Jun 7;15(11):5032–51. doi: 10.18632/aging.204776 (PMC10292893; doi:10.18632/aging.204776)
Supplement: Supplementary Figures [file aging-15-204776-s001.pdf]

SUPPLEMENTARY FIGURES

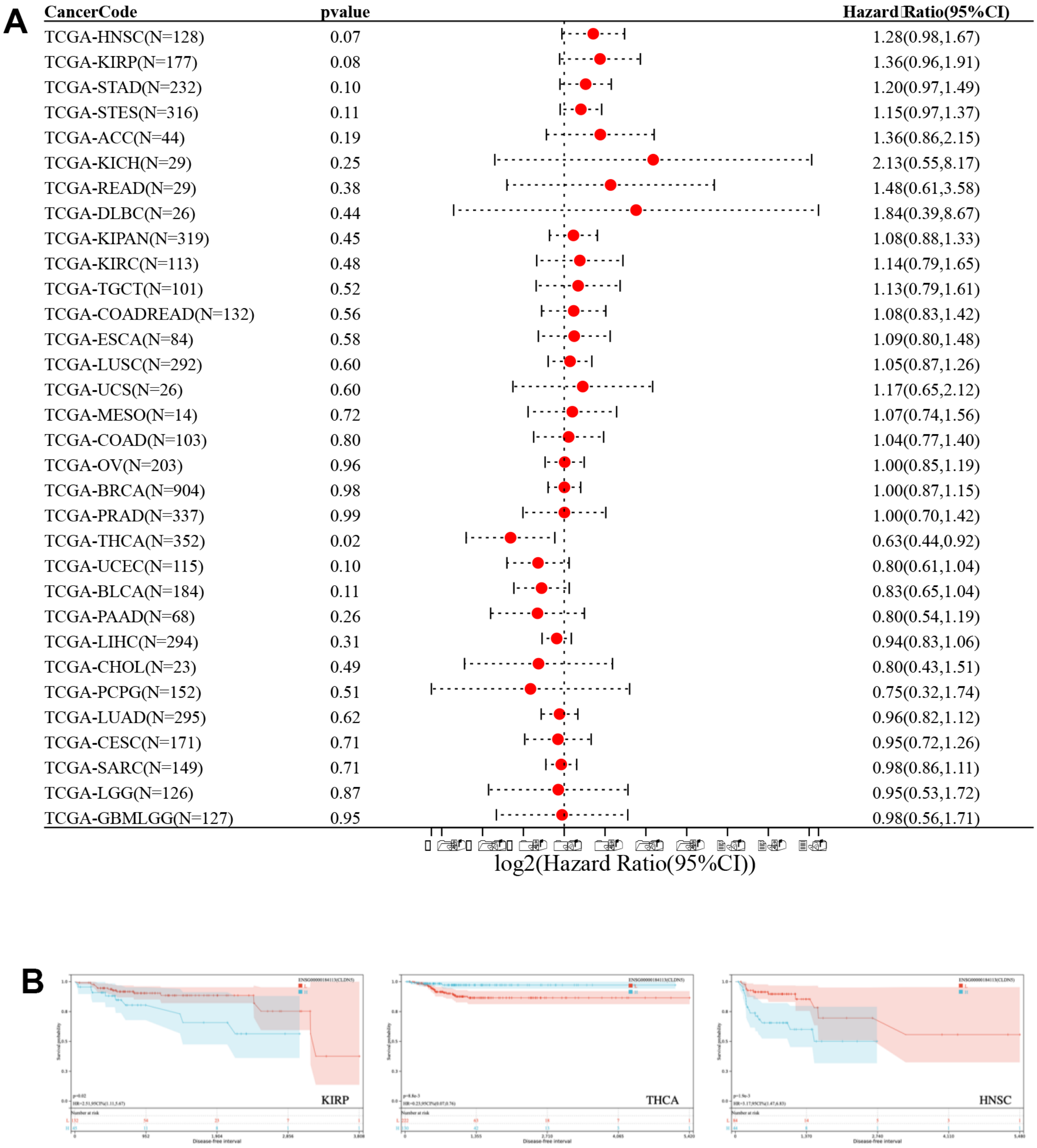

Supplementary Figure 1. Association between CLDN5 expression and PFI of cancer patients. (**A**) A forest plot of hazard ratios of CLDN5 in 36 types of tumors. (**B**) Kaplan-Meier survival curves of PFI for patients stratified by the different expressions of CLDN5 in KIRP, THCA, and HNSC.

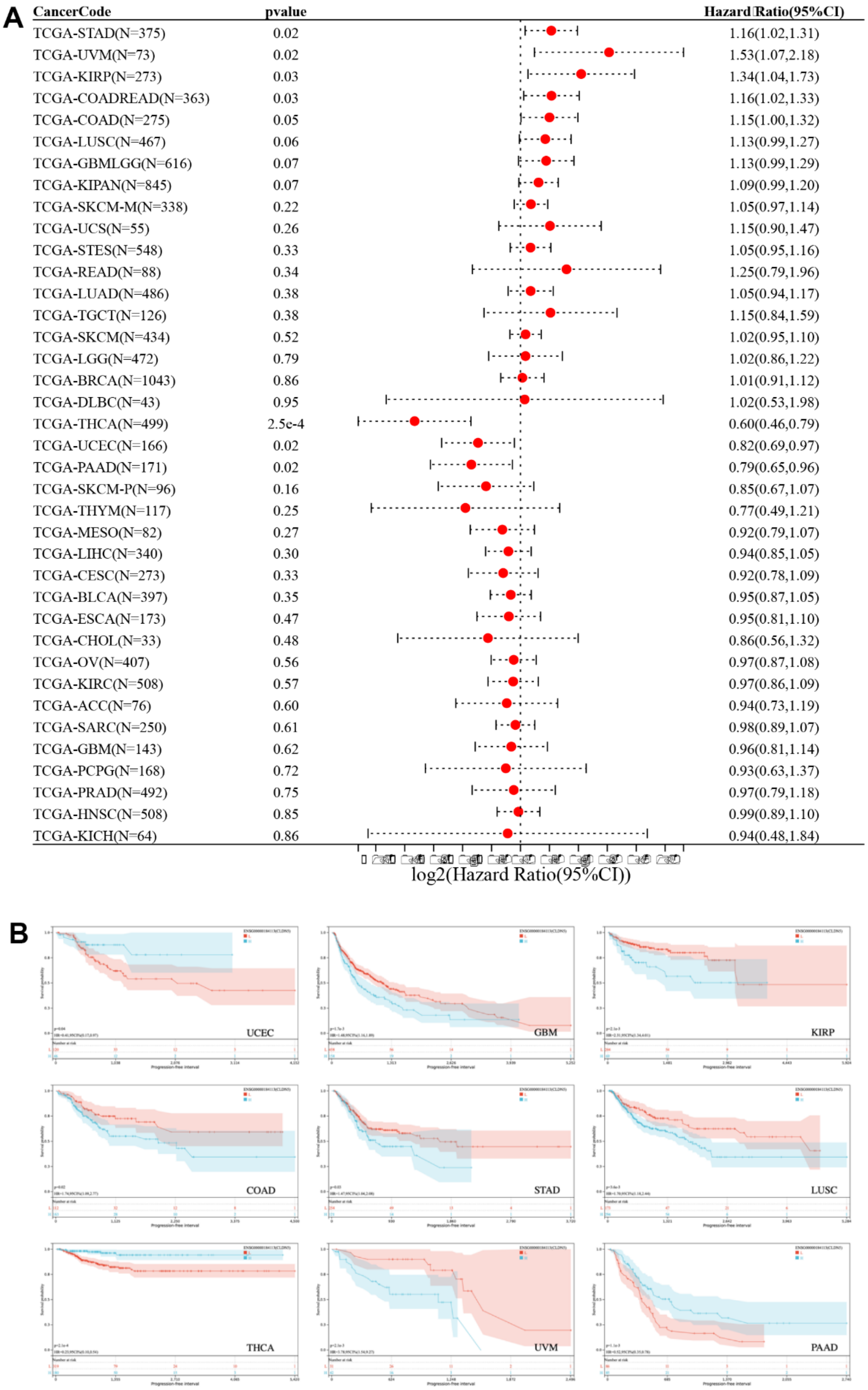

Supplementary Figure 2. Association between CLDN5 expression and DFI in cancer patients. (**A**) A forest plot of hazard ratios of CLDN5 in 36 types of tumors. (**B**) Kaplan-Meier survival curves of DFI for patients stratified by the different expressions of CLDN5 in UCEC, GBM, KIRP, COAD, STAD, LUSC, THCA, UVM, and PAAD.

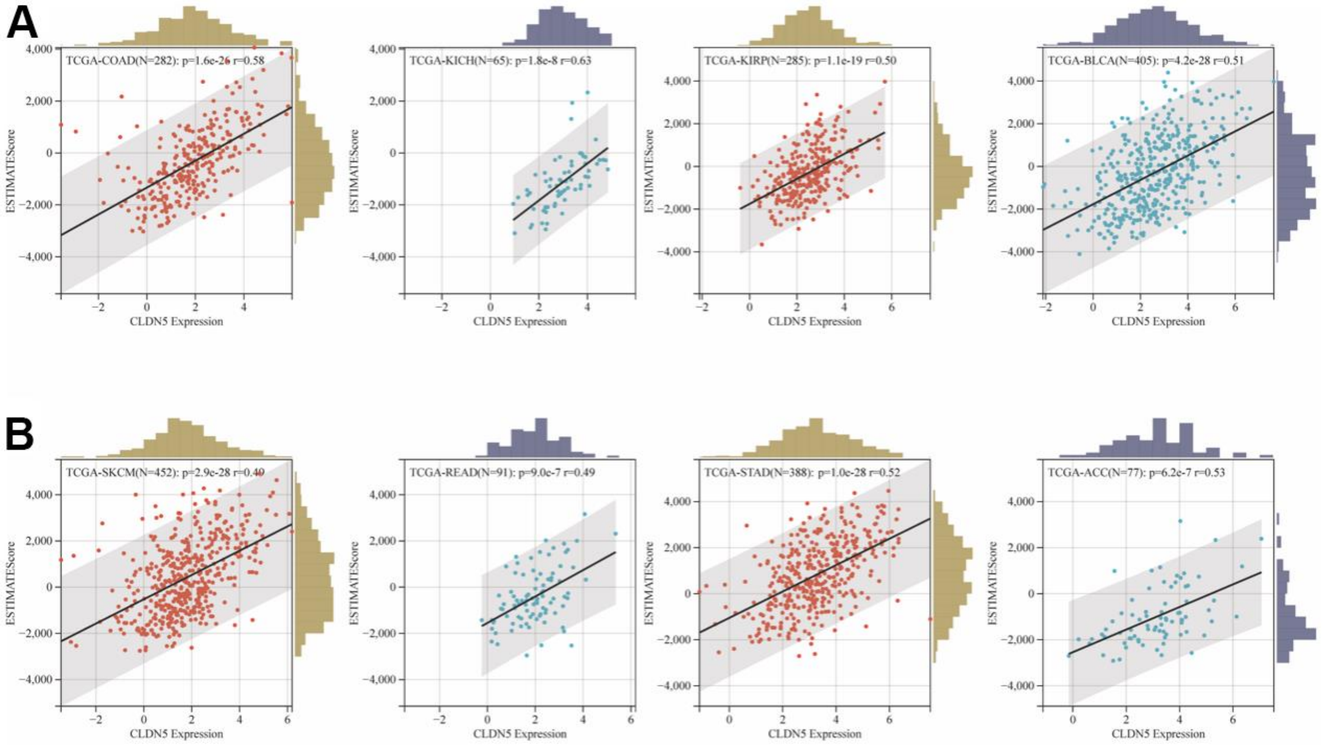

Supplementary Figure 3. Correlation of CLDN5 with ESTIMATESCORE scores. (**A**) The ESTIMATESCORE score in COAD, KICH, KIRP, and BLCA. (**B**) The ESTIMATESCORE score in SKCM, READ, STAD, and ACC.

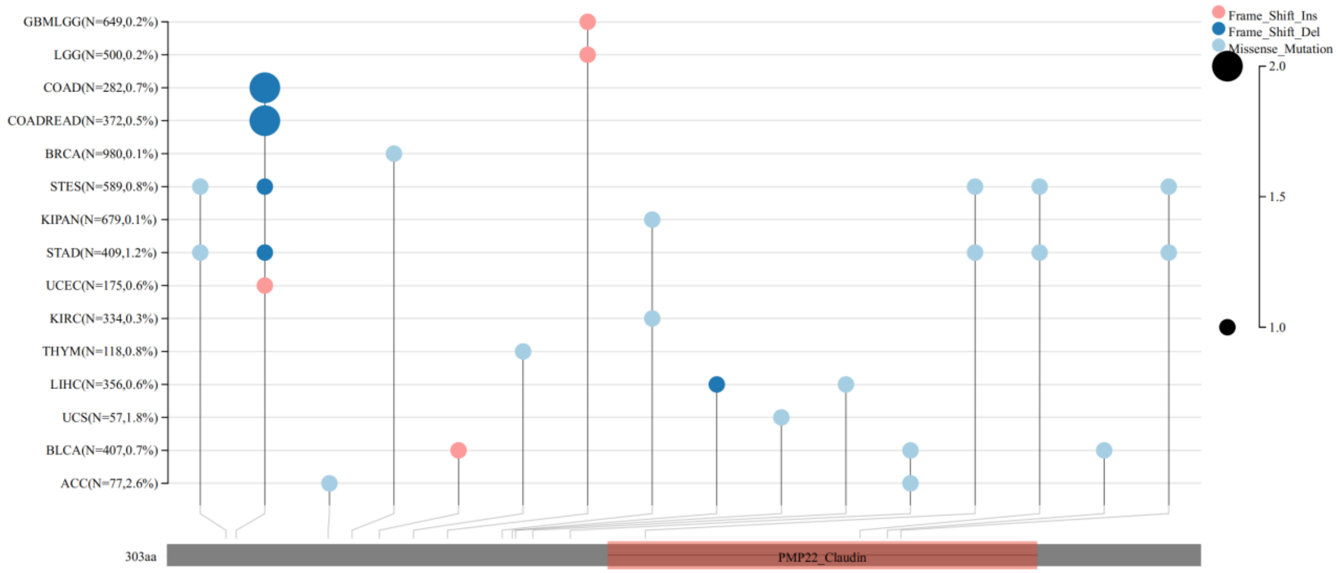

Supplementary Figure 4. The mutational landscape of CLDN5 from TCGA. The frameshift deletion of CLDN5 was markedly in COAD. In STAD, missense mutations of CLDN5 in various sites were obvious.
